# Production of a gonadotropin-releasing hormone 2 receptor knockdown (GNRHR2 KD) swine line

**DOI:** 10.1007/s11248-017-0023-4

**Published:** 2017-05-22

**Authors:** A. T. Desaulniers, R. A. Cederberg, G. A. Mills, C. A. Lents, B. R. White

**Affiliations:** 1 Department of Animal Science, University of Nebraska-Lincoln, A224j Animal Science Building, 3940 Fair Street, Lincoln, NE 68583-0908 USA; 2 USDA, Agricultural Research Service, U.S. Meat Animal Research Center, Clay Center, NE 68933-0166 USA

**Keywords:** GNRH2, GNRHR2, Porcine, Transgenic, Testosterone, Testis, Gene knockdown, shRNA

## Abstract

Swine are the only livestock species that produce both the second mammalian isoform of gonadotropin-releasing hormone (GNRH2) and its receptor (GNRHR2). Previously, we reported that GNRH2 and GNRHR2 mediate LH-independent testosterone secretion from porcine testes. To further explore this ligand-receptor complex, a pig model with reduced *GNRHR2* expression was developed. Small hairpin RNA sequences targeting porcine *GNRHR2* were subcloned into a lentiviral-based vector, lentiviral particles were generated and microinjected into the perivitelline space of zygotes, and embryos were transferred into a recipient. One GNRHR2 knockdown (KD) female was born that subsequently produced 80 piglets from 6 litters with 46 hemizygous progeny (57% transgenic). Hemizygous GNRHR2 KD (n = 10) and littermate control (n = 7) males were monitored at 40, 100, 150, 190, 225 and 300 days of age; body weight and testis size were measured and serum was isolated and assayed for testosterone and luteinizing hormone (LH) concentrations. Body weight of GNRHR2 KD boars was not different from littermate controls (*P* = 0.14), but testes were smaller (*P* < 0.05; 331.8 vs. 374.8 cm^3^, respectively). Testosterone concentrations tended (*P* = 0.06) to be reduced in GNRHR2 KD (1.6 ng/ml) compared to littermate control (4.2 ng/ml) males, but LH levels were similar (*P* = 0.47). The abundance of *GNRHR2* mRNA was reduced (*P* < 0.001) by 69% in testicular tissue from mature GNRHR2 KD (n = 5) versus littermate control (n = 4) animals. These swine represent the first genetically-engineered model to elucidate the function of GNRH2 and its receptor in mammals.

## Introduction

Gonadotropin-releasing hormone (GNRH1; pGlu-His-Trp-Ser-Tyr-Gly-Leu-Arg-Pro-Gly) is well-regarded as the master regulator of reproduction in mammals. A second GnRH isoform (GNRH2; pGlu-His-Trp-Ser-**His**-Gly-**Trp**-**Tyr**-Pro-Gly) has also been identified in mammals (White et al. 1998). Expression of *GNRH2* is ubiquitous (Neill 2002) and is most abundant in tissues outside of the brain (White et al. 1998), suggesting a divergent role from GNRH1. Indeed, GNRH2 is a poor stimulator of gonadotropin release; GNRH2-induced secretion of luteinizing hormone (LH) and follicle-stimulating hormone (FSH) is about 10% that of GNRH1 (Millar et al. 1986).

A 7-transmembrane, G-protein-coupled receptor specific for GNRH2 (GNRHR2) has also been identified in mammals (Millar et al. 2001; Neill et al. 2001). Interestingly, GNRH1 receptor (GNRHR) and GNRHR2 are quite different; their homology is only about 40% and GNRHR2 has an intracytoplasmic tail which GNRHR lacks (Millar 2003). Like its ligand, the *GNRHR2* gene is also expressed ubiquitously, including extra-pituitary reproductive tissues (Millar et al. 2001; Neill et al. 2001). Interestingly, *GNRHR2* mRNA levels were highest in the marmoset testis compared to 31 other organs (Millar et al. 2001). However, most mammalian species are unable to produce a functional GNRHR2 due to gene deletions or gene coding errors (Gault et al. 2004; Millar 2003; Morgan et al. 2003; Neill et al. 2002a; Pawson et al. 2003). In fact, the pig is the only livestock species to maintain the appropriate sequence to encode a full-length GNRHR2 (Stewart et al. 2009). Furthermore, this receptor is considered functional since exogenous GNRH2 treatment stimulated inositol triphosphate production from COS cells overexpressing the porcine GNRHR2 (Neill et al. 2002b).

Studies in boars indirectly suggested that a testicular GnRH isoform mediates steroidogenesis within the porcine testis (Bowen et al. 2006; Wise et al. 2000; Zanella et al. 2000). However, GNRHR is absent in swine testes (Zanella et al. 2000); therefore, we hypothesized that the results of the aforementioned studies could be ascribed to GNRHR2. In support of this hypothesis, the *GNRHR2* gene promoter is active in a swine testis-derived (ST) cell line (Brauer et al. 2016) and both GNRH2 and GNRHR2 protein are abundantly produced within the boar testis (Desaulniers et al. 2015). We also demonstrated that GNRHR2 immunolocalized to porcine Leydig cells and exogenous GNRH2 treatment stimulated LH-independent testosterone secretion from testes of boars (Desaulniers et al. 2015). These data suggest that swine maintain a unique testicular GNRH2-GNRHR2 system capable of regulating testosterone secretion in a paracrine manner that is independent from the classical androgen stimulator, LH. However, the function and cellular mechanisms of GNRH2-mediated testosterone secretion remain unknown. Thus, we developed a GNRHR2 knockdown (KD) swine line to further examine the role of GNRH2 and its receptor in the boar testis.

## Materials and methods

### Animals

White crossbred pigs from the University of Nebraska-Lincoln (UNL) Agricultural Research and Development Center (ARDC) swine unit (Mead, NE) were used to generate the founder animal. Yorkshire boar semen, purchased from Swine Genetics International (Cambridge, IA), was used to produce progeny from the founder female. Farrowing and subsequent housing occurred within the UNL Animal Science Building (Lincoln, NE). Pre-pubertal pigs were group housed with *ad libitum* access to feed and water. Adult pigs were housed individually with *ad libitum* access to water and fed approximately 2.5 kg of feed daily. All diets were formulated to meet National Research Council guidelines for each stage of production.

### Vector preparation, production of lentiviral particles and ST cell transduction

Utilizing oligomer design software (Clontech, Mountain View, CA), two potential target small hairpin (shRNA) sequences, shRNA1 (TGGCTACTCAGTTTCCTTC) and shRNA2 (CTGTCATGACCATCTGCTA), specific to the porcine *GNRHR2* (NCBI accession number NM_001001639.2) were identified and subcloned into a lentiviral-based, pLVX-shRNA2 vector (Cat # 632179; Clontech) that provides both ubiquitous shRNA and fluorescent ZsGreen1 co-expression. Neither shRNA sequences matched other genes in the porcine genome including *GNRHR* (NCBI accession number JN120792.1). The shRNA expression was driven by the human U6 promoter, whereas ZsGreen1 expression was controlled by the cytomegalovirus (CMV) promoter. Lentiviral particles were produced from each shRNA vector, as well as a control vector, by transfection with Fugene6 (Promega, Madison, WI) and the Lenti-X HTX Packaging Mix into Lenti-X 293T cells (Clontech). Briefly, 4 × 10^6^ cells were plated in 10 cm dishes at approximately 70% confluency, and cells were transfected with 5 µg of vector, 15 µl of Lenti-X HTX Packaging Mix (Clontech) and 30 μl Fugene6 (Promega). Approximately 48 h later, particles were harvested and concentrated (100X) with Lenti-X Concentrator (Clontech) according to the manufacturer’s instructions. Titrations of viral particles were performed by infecting swine testis-derived (ST) cells (CRL-1746; ATCC, Manassas, VA) with serial dilutions of lentivirus and counting fluorescent cells to determine infectious units (IU)/ml.

The ability of shRNA1 and shRNA2 lentiviral particles to ablate porcine *GNRHR2* mRNA levels was tested in ST cells. The day prior to transduction, ST cells (1.2 × 10^6^ cells/plate) were plated in 100 mm plates containing high-glucose Dulbecco’s Modified Eagle’s Medium (Mediatech, Herndon, VA) supplemented with 10% FBS, 2 mM glutamine, 100 U/ml penicillin, and 100 μg/ml streptomycin sulfate (Gibco, Grand Island, NY). Cells were transduced with 1.44 × 10^7^ viral particles per plate for 48 h. At 48 h post-transduction, RNA was isolated from ST cells with TRIzol (1.5 ml/plate; Invitrogen, Grand Island, NY) per manufacturer’s instructions and stored at −20 °C until reverse transcription (RT).

### Quantitative PCR

Ribonucleic acid from ST cells was DNase treated with RQ1 (Promega) and quantitated using a Nanodrop spectrophotometer (NanoDrop Technologies, Inc., Wilmington, DE). The RNA was reverse transcribed into cDNA with M-MLV (Promega) and random hexamers (Promega). The PCR amplification of *GNRHR2* was performed using forward (F-CCCCGGACAAGGAAGGG) and reverse (R-AAGGAGCGACGGAGGGTCAA) primers (Integrated DNA Technologies, Coralville, Iowa) as well as a FAM labeled TaqMan MGB probe (ATGATGCCCCTGCCGG; Applied Biosystems, Foster City, CA). Quantitative PCR was performed on cDNA samples with an ABI 7900 HT instrument (Applied Biosystems). The amplification protocol included an initial 50 °C step for 2 min followed by 95 °C for 10 min and then alternating temperatures between 95 °C for 15 s and 60 °C for 1 min, repeated 50 times. The housekeeping gene was eukaryotic *RNA18S* (kit #4319413E; VIC/MGB Probe; Applied Biosystems). Cycling conditions for *RNA18S* were the same as those described for *GNRHR2*. Internal controls included samples prepared without reverse transcriptase (no RT control) and without cDNA (water only control).

### Generation of transgenic pigs

RNA interference (RNAi) has successfully been employed to impair gene expression in a variety of mammalian cells (Dann 2007; Park 2007; Roelz et al. 2010; Rubinson et al. 2003). Transducing zygotes via viral particles has generated transgenic mice (Miao et al. 2011; Rubinson et al. 2003), rats (Dann 2007), chickens (McGrew et al. 2004), goats (Golding et al. 2006) and pigs (Hofmann et al. 2003; Whitelaw et al. 2004). In this study, we utilized lentiviral particles and RNAi to produce a transgenic model to study the function of GNRHR2 in swine. Briefly, zygotes (n = 50) were surgically flushed from the oviduct of one superovulated, white crossbred donor sow. Embryos were collected in Beltsville embryo culture medium (BECM; 19.3 mM sodium lactate, 2.13 mM calcium lactate, 90 mM sodium chloride, 4.83 mM potassium chloride, 0.54 mM magnesium chloride, 2.14 mM sodium bicarbonate, 10.91 mM Hepes free acid, 0.55 mM glucose, 11 mM mannitol, 1 mM l-glutamine, 7 mM taurine, 0.27 mM sodium pyruvate, 80 mM EDTA free acid, 0.001% phenol red, 1% BSA, 50 μg gentamycin sulfate; Dobrinsky et al. 1996) and cultured briefly (1 h) prior to microinjection. All reagents for BECM were purchased from Sigma (St. Louis, MO) except l-glutamine, EDTA and gentamycin sulfate (Gibco). Embryos were microinjected with lentiviral particles according to a protocol generously shared by Dr. Charles Long (Texas A&M University, College Station, TX). Briefly, embryos were placed in 100 µl microdrops of BECM + 200 mM sucrose (Fisher, Fair Lawn, NJ) on micromanipulation plates (100 × 15 mm petri dish; VWR International, Batavia, IL) layered with embryo-tested mineral oil (M8410; Sigma). Sucrose was utilized to retract the cytoplasm from the zona pellucida to facilitate microinjection into the perivitelline space (Miao et al. 2011). Using a holding pipet (VacuTip; ID = 15 µm; OD = 100 µm; Eppendorf, AF, Hamburg, Germany) and an intracytoplasmic sperm injection (ICSI) needle (TransferTip-F; ID = 4 µm; OD = 7 µm; Eppendorf) backloaded with 5 µl of lentiviral particles (1.15 × 10^9^ IU/ml), each embryo was microinjected within the perivitelline space for 30 s using the manual setting (pi = 150 hPa; pf = 0 hPa) on the Eppendorf Femtojet injection system with a Nikon diaphot inverted microscope (Melville, NY). A total of 40 microinjected zygotes were surgically transferred into the oviduct of one synchronized recipient female, which was allowed to gestate to term.

### Confirmation of transgene integration

Upon birth of piglets from the recipient, expression of ZsGreen1 was examined with a UV light and a Roscolux #15 (deep straw; Rosco, Port Chester, NY) filter. In addition, genomic DNA was isolated from tail samples using a DNeasy Blood and Tissue Kit (Qiagen, Valencia, CA). Transgene integration was evaluated via conventional PCR using primers designed to amplify a portion of the human U6 promoter driving the shRNA (F-GAGGGCCTATTTCCCATGAT; R-GATCCTCGTCCTTTCCACAA), a region from the U6 promoter to the multiple cloning site (U6/MCS) to verify incorporation of the shRNA sequence (F- GAGGGCCTATTTCCCATGAT; R-GGGCGTACTTGGCATATGAT), and a portion of the ZsGreen1 coding sequence (F-CTGCATGTACCACGAGTCCA; R-GTCAGCTTGTGCTGGATGAA). The Philisa Thermocycler (Streck Inc., Omaha, NE) was used for rapid evaluation of samples with the following conditions for both the U6 and ZsGreen1 reactions: 1 X *Taq* Buffer A (Fisher) with a final concentration of 3.0 mM MgCl_2_, 200 µM dNTPs, 400 nM of each primer and 1.25 U of *Taq* DNA Polymerase (Fisher). Cycling conditions were 95 °C for 60 s, followed by 35 cycles of 95 °C for 5 s, 55 °C for 5 s and 72 °C for 10 s and a final extension of 72 °C for 20 s. The U6/MCS PCR reaction conditions were the same, however the cycling conditions were as follows: 95 °C for 60 s, followed by 35 cycles of 95 °C for 10 s, 56 °C for 10 s and 72 °C for 15 s and a final extension of 72 °C for 30 s. The resultant PCR products were subjected to electrophoresis on a 1% agarose gel.

Inverse PCR was performed as described previously (Cederberg et al. 2015) to determine the location and number of integration sites. Briefly, genomic DNA was digested with *Xba*I or *Hind*III and fragments were self-ligated with a high concentration of T4 DNA ligase (0.1 IU/µl; Thermo Scientific Fermentas, Pittsburg, PA) and a low concentration (2 µg/µl) of DNA. PCR was performed on the circularized DNA with primers selected from the integrated pLVX-shRNA2 sequence (*Xba*I-F: GAGATCCCTCAGACCCTTTT, *Xba*I-R: GTTGCGTCAGCAAACACAGT; *Hind*III-F: GAGCCCTCAGATCCTGCATA, *Hind*III-R: AGCACCATCCAAAGGTCAGT) and in reverse orientation of typical primers. Resultant PCR products were sequenced with nested primers for *Xba*I (TCCCTCAGACCCTTTTAGTCA) and *Hind*III (CTGGCCCTGGTGTGTAGTTC) derived products at the University of Nebraska Medical Center Genomic Core Facility (Omaha, NE).

### Characterization of GNRHR2 KD swine line

At 40, 100, 150, 190, 225 and 300 days of age, testis size of GNRHR2 KD (n = 10) and littermate control (n = 7) boars was measured using digital calipers. Predicted testis volume was calculated using the following equation: Volume = 3/4(π)(L/2)(W/2)^2^, where L = testis length and W = testis width (Bailey et al. 1998). Body weight was recorded and blood samples were collected via jugular venipuncture. Blood samples were allowed to clot at 4 °C overnight before centrifugation (2000 × *g*). Serum was collected and stored at −20 °C until use. Transgenic (n = 5) and littermate control boars (n = 4) were subsequently euthanized (717 ± 24 days of age) by intravenous injection of Fatal-Plus Solution (1 mg/4.5 kg; pentobarbital; Vortech Pharmaceuticals, Dearborn, MI). Testicular tissue was collected immediately and stored in RNA*later* (100 mg/ml; Qiagen) at −20 °C for subsequent mRNA analysis.

### Radioimmunoassay

Unextracted serum was analyzed in duplicate to quantify concentrations of testosterone by a double antibody radioimmunoassay (RIA) kit (cat # 07-189102; MP Biomedicals, Aurora, OH), in accordance with manufacturer’s instructions. Additional standards (0.03 and 0.16 ng/ml) were added to reach the sensitivity of the assay (0.03 ng/ml). The intra- and inter-assay coefficients of variation (CV) were 6.5 and 7.7%, respectively. Concentrations of LH were determined by RIA at the Colorado State University Reproductive Endocrinology Laboratory (Fort Collins, CO). The intra- and inter-assay CV were 2.7 and 6.2%, respectively.

### Digital droplet PCR

Digital droplet PCR (ddPCR) was used to quantify the abundance of *GNRHR2* mRNA in testes of transgenic (n = 5) and control (n = 4) boars. Samples were removed from RNA*later* and homogenized in TRIzol (1 ml; Invitrogen) with a Tissue Tearor (Biospec Products Inc., Bartlesville, OK). Total RNA was subsequently extracted using standard procedures with chloroform and isopropyl alcohol. Ribonucleic acid was DNAse treated, quantitated on a Nanodrop spectrophotometer (NanoDrop Technologies, Inc.) and reverse transcribed (2 ug) into cDNA as described above.

To quantify *GNRHR2* transcript levels, specific primers (Integrated DNA Technologies) and a FAM labeled TaqMan MGB probe (Applied Biosystems) were utilized as described above. Each reaction contained 1 µl of the cDNA template (undiluted), 900 nM of each primer, 250 nM of the probe and ddPCR Supermix for Probes (Bio-Rad, Richmond, CA). Droplets were generated using the QX200 Droplet Generator (Bio-Rad) according to manufacturer’s instructions. A C1000 Touch Thermal Cycler (Bio-Rad) was utilized with the following conditions: 95 °C for 5 min (enzyme activation), 95 °C for 30 s (denaturation) followed by 60 °C for 1 min (annealing/extension; 40 cycles), 4 °C for 5 min and 90 °C for 5 min (signal stabilization). Droplets were read via the QX200 droplet reader (Bio-Rad) and analyzed using Quantasoft Software (Bio-Rad). The housekeeping gene utilized was *ACTB*; each reaction contained 1 µl of the cDNA template (diluted 1:100), 100 nM of each primer (F-AACTCCATCATGAAGTGCGACG; R-GATCCACATCTGCTGGAAGG) and EvaGreen ddPCR Supermix (Bio-Rad). Cycling conditions for *ACTB* were the same as those described for *GNRHR2* reactions.

### Statistical analysis

Statistical analyses were performed using the Statistical Analysis System (SAS; Cary, NC). The mRNA data was analyzed using the MIXED procedure of SAS with lentiviral treatment or swine line included in the model as the fixed effect. When applicable, litter was included in the model as a random effect. The experimental unit was either plate or animal. When a significant treatment effect (*P* ≤ 0.05) was observed, pair-wise comparisons were made using the Tukey-Kramer test.

Hormone data, predicted testis volume and body weight were analyzed via the MIXED procedure of SAS using a model that included line (transgenic or control), age (days), and their interaction as fixed effects. Animal was the experimental unit and litter was included as a random effect. Age was used as the repeated measure with animal as the subject. Degrees of freedom for the pooled error term were calculated using the Satterthwaite approximation. Based on the Akaike’s information criterion, a heterogeneous autoregressive function with lag equal to 1 was used to model the covariance structure for the repeated measures. When analyzing predicted testis volume, body weight was found to be a significant covariable (*P* = 0.02) and therefore was included in the statistical model. In contrast, predicted testis volume was not found to be a significant covariable for testosterone concentration, therefore it was excluded from the analysis. A single LH value (control boar; 150 days of age) was found to be an outlier (± 2 standard deviations from the mean) so it was excluded from the dataset. A *P* value of ≤ 0.05 was considered significant, whereas a *P* value of ≤ 0.10 was considered a tendency. Results are presented as least squares means (LSMEANS) ± the standard error of the mean (SEM).

## Results and discussion

**Fig. 1:**
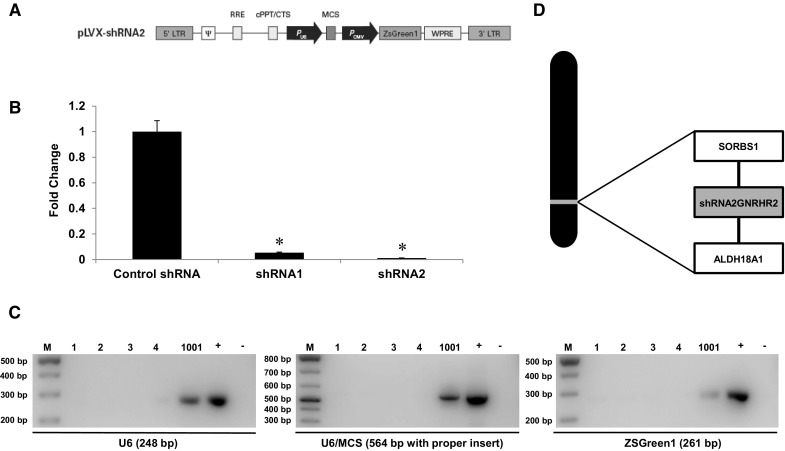
Generation of GNRHR2 KD pigs. **a** Schematic representation of the construct used for lentiviral particle generation. **b** *GNRHR2* mRNA levels in ST cells following transduction with lentiviral particles containing shRNA specific for the GNRHR2 or control. Data are presented as the LSMEANS ± SEM. *P < 0.05. **c** PCR detection of GNRHR2 KD founder (#1001) and control littermates (#1–4). M, DNA ladder; +, positive plasmid control; −, negative control. **d** Sequencing analysis of inverse PCR products revealed that a single integration site was present on chromosome 14, located between the Sorbin and SH3 Domain Containing 1 (*SORBS1*) and the Aldehyde Dehydrogenase 18 Family, Member A1 (*ALDH18A1*) genes

Previously, our laboratory detected GNRHR2 protein within the swine testis (Desaulniers et al. 2015) as well as the ST cell line (data not shown). Therefore, we transduced ST cells with lentiviral particles derived from a vector (Fig. 1a) overexpressing shRNA specific to the GNRHR2 (shRNA1 and shRNA2) in order to test the efficacy of lentiviral particles prior to production of a transgenic pig line. Lentiviral particles containing either shRNA1 or shRNA2 sequences significantly reduced *GNRHR2* mRNA levels (95 and 99%, respectively) compared to control particles (*P* < 0.05; Fig. 1b). After the efficiency of the lentiviral particles was confirmed in vitro, lentiviral particles derived from the vector overexpressing shRNA2 were microinjected into porcine zygotes before transfer into a synchronized recipient female.

The recipient gestated for 113 days and farrowed 5 healthy, live piglets (2 males and 3 females). One female exhibited ZsGreen1 fluorescence of the skin (20% efficiency), indicative of successful transgene integration and expression. Transgene integration was confirmed via conventional PCR using primers designed to amplify the human U6 promoter driving the shRNA, ZsGreen1, and a region from the U6 promoter to the multiple cloning site (U6/MCS) to verify incorporation of the shRNA sequence (Fig. 1c). Sequence analysis of products from inverse PCR procedures revealed that a single integration site was present on chromosome 14, aligning with clone NW_003612067.1 with 99% identity and matching identities 448,946-448,372. Thus, the transgene integrated in a non-coding region between the Sorbin and SH3 Domain Containing 1 (*SORBS1*) and the Aldehyde Dehydrogenase 18 Family, Member A1 (*ALDH18A1*) genes (Fig. 1d). This animal was retained to generate the GNRHR2 KD swine line.

The founder gilt was bred at her third post-pubertal estrus to a commercial white crossbred boar and subsequently gestated to term. She farrowed 15 healthy piglets with 5 progeny exhibiting ZsGreen1 expression (3 males and 2 females). The founder female subsequently produced a total of 80 piglets from 6 litters (6 different white crossbred sires) with 46 transgenic piglets (57%), confirming germline transmission of the transgene and providing further evidence for a single integration site.

**Fig. 2:**
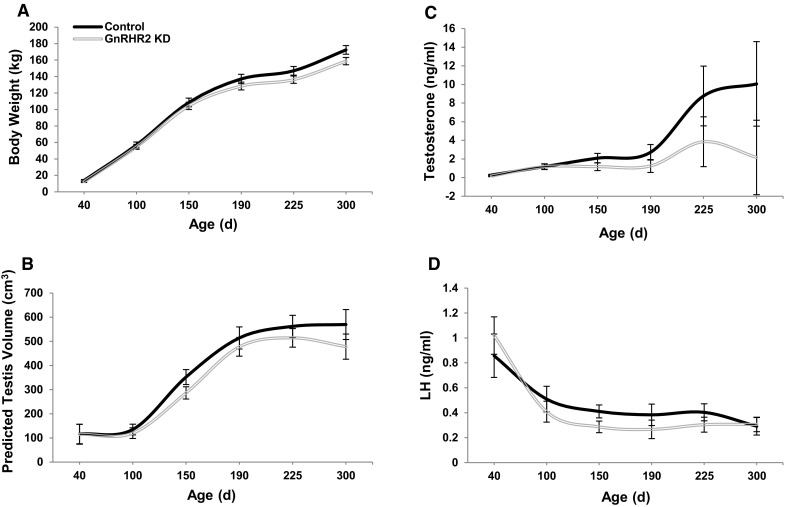
GNRHR2 KD swine (n = 10) have smaller testes and reduced serum testosterone concentrations compared to littermate control (n = 7) animals during pubertal development. At 40, 100, 150, 190, 225 and 300 days of age, blood was collected and body weight and predicted testis volume were determined. Serum testosterone and LH concentrations were determined by radioimmunoassay. Data are presented as the LSMEANS ± SEM. **a** Body weight: Line, *P* = 0.14; Time, *P* < 0.0001; Line × Time, *P* = 0.61. Predicted testis volume (**b**): Line, *P* = 0.05; Time, *P* < 0.0001; Line × Time, *P* = 0.39. Testosterone (**c**), Line, *P* = 0.06; Time, *P* < 0.0001; Line × Time, *P* = 0.39. Luteinizing hormone (**d**), Line, *P* = 0.47; Time, *P* < 0.0001; Line × Time, *P* = 0.52

During pubertal development, hemizygous GNRHR2 KD (n = 10) and littermate control (n = 7) boars from 4 litters were monitored for differences in body weight, predicted testis volume and serum hormone (testosterone and LH) concentrations. Body weight was not affected by line (*P* = 0.14) or line by age interaction (*P* = 0.61), indicating that GNRHR2 KD and littermate control boars had a similar body weight during pubertal development (Fig. 2a). Despite a similar body weight, there was a line effect (*P* < 0.05) for predicted testis volume. Predicted testis volume of GNRHR2 KD boars was smaller (331.77 ± 13.9 cm^3^) overall during pubertal development than for littermate controls (374.75 ± 17.17 cm^3^; Fig. 2b), suggesting a defect in testis development. As expected, there was an effect of age on predicted testis volume (*P* < 0.0001) but there was no line by age interaction (*P* = 0.39; Fig. 2b).

**Fig. 3:**
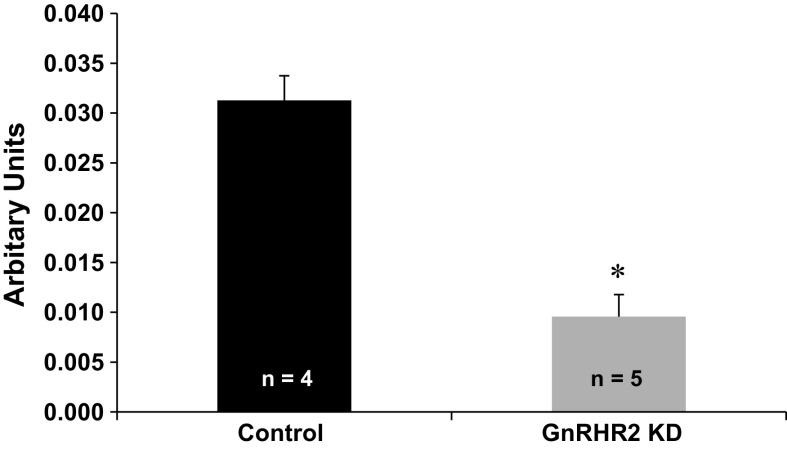
Relative *GNRHR2* mRNA levels are significantly reduced within the testes of GNRHR2 KD versus littermate control boars. Digital Droplet PCR revealed a 69% reduction in *GNRHR2* mRNA levels (normalized to *ACTB* mRNA levels) in the testes of transgenic (n = 5) compared to littermate control (n = 4) boars. Data are presented as the LSMEANS ± SEM. *P < 0.001

There was no line by age interaction (*P* = 0.39; Fig. 2c) on concentration of testosterone in serum. However, there was a tendency for a line effect (*P* = 0.06; Fig. 2c) as well as an expected effect of age (*P* < 0.0001; Fig. 2c). GNRHR2 KD boars tended to have reduced concentrations of testosterone in serum overall during pubertal development (1.63 ± 0.87 ng/ml) compared with littermate control animals (4.18 ± 1.0 ng/ml; *P* = 0.06). Despite differences in testosterone secretion, LH concentrations were similar between GNRHR2 KD and control boars during development (*P* = 0.47; Fig. 2d). To confirm knockdown in these animals, transgenic (n = 5) and control (n = 4) testicular tissue was collected and *GNRHR2* mRNA abundance was determined via ddPCR. Testicular *GNRHR2* mRNA levels in transgenic boars were approximately 69% less than littermate control boars (*P* < 0.001; Fig. 3), confirming the successful production of a GNRHR2 KD swine line. These findings are consistent with our previous report demonstrating that GNRH2 stimulates testosterone secretion *in vivo*, without the classic preceding rise in serum LH levels associated with GNRH1 (Desaulniers et al. 2015) and strengthen our hypothesis that GNRHR2 is involved in testicular steroidogenesis within swine testes. However, since LH and testosterone are released in a pulsatile manner, it will be prudent to collect serial blood samples as well.

In conclusion, these swine represent the first genetically-engineered animal model to unravel the biological function of GNRH2 and its receptor in mammals. During pubertal development, GNRHR2 KD boars had smaller testes despite similar body weights. Transgenic males also tended to have reduced serum testosterone concentrations, independent of alterations in circulating LH levels. These results indicate that GNRHR2 is directly involved in the regulation of testicular steroidogenesis in swine. This animal model will be used in future studies to elucidate the cellular mechanisms associated with localized regulation of testosterone secretion and define what consequences it may have for fertility in pigs.
